# Re-participation intention in individuals playing tennis for recreational purposes: investigation of differences based on low and high involvement

**DOI:** 10.3389/fpsyg.2025.1546405

**Published:** 2025-03-10

**Authors:** Omur Fatih Karakullukcu, Cihan Ayhan, Tugcenur Kalkan, Bilal Okudan, Laurentiu-Gabriel Talaghir, Teodora Mihaela Iconomescu

**Affiliations:** ^1^Ministry of National Education, Ankara, Türkiye; ^2^Department of Recreation, Sakarya University of Applied Sciences, Sakarya, Türkiye; ^3^Graduate Education Institute, Sakarya University of Applied Sciences, Sakarya, Türkiye; ^4^Independent Researcher, Ankara, Türkiye; ^5^Faculty of Physical Education and Sport, Dunarea de Jos University of Galati, Galati, Romania

**Keywords:** tennis, involvement, recreation, racket sports, re-participation intention

## Abstract

**Purpose:**

This study examined how the level of involvement influences the re-participation intentions of recreational tennis players.

**Method:**

To measure the participants’ leisure involvement levels; the leisure involvement scale, developed by Kyle et al. (2007) and adapted to the Turkish language by Gürbüz et al. (2018) was used. The behavioral intentions scale developed by Zeithaml et al. (1996) was used to measure participants’ re-participation intentions. The research group consists of a total of 327 volunteer individuals, 172 males (52.6%) and 155 females (47.4%), selected by convenience sampling method among individuals who play tennis for recreational purposes. The normality test of the data obtained was tested by checking the skewness-kurtosis values and it was determined that the data showed normal distribution. In this context, in addition to descriptive statistics, Hierarchical-Non-Hierarchical Clustering Analysis, and Univariate analyses were used in the analysis of the data.

**Results:**

According to the research findings, it was determined that there was a statistically significant difference in the re-participation intentions of the participants who played tennis for recreational purposes according to their involvement levels.

**Conclusion:**

It was observed that participants with high involvement levels had higher re-participation intentions than those with low involvement.

## 1 Introduction

In today’s fast-paced life, individuals need to spend their leisure time engaging in activities they enjoy, as it contribute to both physical and mental wellbeing. For those who play tennis recreationally, this becomes even more meaningful due to the benefits it offers. Such recreational activities not only enhance physical fitness but also provide an opportunity to relieve stress and achieve mental relaxation. Regular participation in these activities is significantly influenced by individuals’ leisure involvement (Chen, 2001). Social interactions, enjoyable experiences, and suitable environments can also serve as motivating factors for tennis players to continue the sport. These factors contribute to forming a sustainable habit by increasing the desire to participate in tennis, driven by high levels of involvement.

In consumer behavior research, the concept of “involvement” was initially developed to evaluate the effects of advertising on individuals. Over time, it has become a key term used to explain consumers’ connections with and interest in specific products or services (Krugman, 1977). According to Mitchell (1979), involvement is defined as a personal state variable that changes based on an individual’s intrinsic motivation and the level of interest triggered by a specific stimulus or situation. This concept is viewed as a psychological state reflecting an individual’s level of interest and arousal toward a particular subject. Later, the concept was adopted in the leisure context, where leisure involvement is defined as an unobservable state of motivation, arousal, or interest in a recreational activity (Chang and Gibson, 2011; Havitz and Dimanche, 1997). The literature divides leisure involvement into two categories: behavioral involvement and psychological involvement (Pan et al., 2018). Behavioral involvement refers to the time and energy individuals devote to specific activities (Stone, 1984), while psychological involvement describes the internal psychological process that drives individuals to participate in leisure activities (Chang, 2017).

Leisure involvement is closely associated with the frequency of participation in activities (Pan et al., 2018; Sato et al., 2017) and significantly influences participants’ re-participation intention (Chen and Shao, 2006). As participants experience increased levels of excitement and enjoyment through consistent participation, their willingness to engage in activities grows as they overcome participation barriers (Gumus et al., 2024; Lin and Feng, 2015). Participation refers to an individual’s attitude toward activities, with the potential to influence and apply their understanding and decision-making abilities (Josiam et al., 1999). Consumers’ responses to the events they participate in provide crucial insights into behavioral patterns. Specifically, re-participation intention signifies the tendency of individuals to return to a previously experienced product or service based on their motivation level. This tendency plays a critical role in identifying factors that influence consumption decisions.

Individuals’ interest in leisure activities and the enjoyment they derive from them can lead to positive changes in their re-participation intentions. This suggests that individuals are inclined to achieve higher psychological satisfaction when engaging in leisure activities. Furthermore, it has been observed that individuals with higher participation levels exhibit more diverse and complex behavioral outcomes (Ahn and Kwon, 2019). This indicates that individuals with a high participation profile demonstrate more sophisticated consumer preferences. In this context, individuals with varying levels of interest in sports display different decision-making models and attitudes when selecting sports activities, which, in turn, creates variations in their willingness to re-participate (Chen and Shao, 2006; Chao-Sen, 2018). In this context, the re-participation intentions of individuals engaging in tennis activities are shaped by similar dynamics.

Tennis, with its individual and competitive aspects, is a sport that can trigger different motivations (Unierzyski, 2003). For instance, individuals who play tennis may exhibit a tendency for re-participation based on factors such as their interest in the technical and tactical aspects of the game, the physical and mental benefits of physical activity, and social interaction (Bum and Jeon, 2016; Spring et al., 2020; Stubbs and Werneck, 2024). Particularly for individuals who play tennis for recreational purposes, the level of satisfaction derived from the activity can significantly influence their re-participation intentions. Those who have had a positive experience are more likely to develop higher levels of involvement, leading them to re-engage in tennis activities due to the enjoyment and psychological satisfaction they gain from the sport. Moreover, individuals’ frequency of play and skill levels are among the key variables determining their re-participation intentions (Casper et al., 2007; Stubbs and Werneck, 2024). Individuals who play tennis regularly and experience skill development during this process are more likely to increase their commitment to the sport, ultimately exhibiting a stronger tendency for long-term re-participation. Consequently, the positive outcomes obtained from tennis activities strengthen the individual’s engagement with the sport, making their consumer behaviors related to re-participation intentions more complex.

Within the framework of the theory of planned behavior, an individual’s prior planning of a specific behavior and possessing strong motivation toward that behavior can enhance their involvement in the activity, thereby increasing their re-participation intention (Burnkrant and Sawyer, 1983). The impact of leisure activities on individuals becomes more significant when participants show intense interest in these activities. This intense engagement, characterized by high levels of participation, continuity, and commitment, allows the activity to become an essential and central part of their lives (Zaichkowsky, 1985; Kyle and Chick, 2002). When evaluated within the framework of the Theory of Planned Behavior, the re-participation intentions of individuals engaging in tennis activities are shaped by their level of involvement in the sport. Individuals who play tennis tend to exhibit re-participation intention by making proper plans and demonstrating a high level of involvement in the activity. In light of this information, it can be stated that leisure involvement is a significant predictor of re-participation intention among tennis participants. In this context, the hypothesis of the study is formulated as follows:

H: The re-participation intention of individuals playing tennis for recreational purposes varies depending on their level of leisure involvement.

In line with this hypothesis, this study aimed to investigate the re-participation intentions of individuals who play tennis for recreational purposes based on their levels of involvement.

## 2 Methods

### 2.1 Research model

This study was based on a quantitative research design. In a population consisting of a large number of elements, the general survey model has been applied to draw general conclusions about the population through a census or a sample selected from it (Karasar, 2012). This study, aimed at investigating the re-participation intention based on participants’ levels of involvement, is designed according to the relational model, which is one of the quantitative research methods.

### 2.2 Research group

The data for this study are planned to be collected from individuals who participate in recreational tennis. Due to the challenges of reaching all participants in terms of time and cost, and the inability to obtain a clear count of individuals involved in recreational tennis activities in Turkey, it has been decided to limit the study group using a purposive sampling method. In this context, 327 individuals who participated in recreational tennis activities in Sakarya province volunteered to participate in the study. After a preliminary review of the data, 73 surveys with incomplete, incorrect, or repeated responses were excluded from the analysis. Therefore, the data from a total of 327 participants (172 males and 155 females, [ $\bar{Χ}$ _Age_ = 21.95 ± 2.44)] were included in the analysis. The demographic characteristics of the participants were presented in Table 1.

**Table 1:** Demographic characteristics of participants.

Age	Mean	S.D.
	21.95	2.44
Gender	*F*	%
Male	172	52.6
Female	155	47.4
Education level	*F*	%
Secondary education	17	5.2
Bachelor’s degree	297	90.8
Graduate degree	13	4.0
Purpose of participation	*F*	%
Socialization	166	49.2
Physical health	122	37.3
Mental health	44	13.5
Participation frequency	*F*	%
Once a week	185	56.6
Twice a week	103	31.5
≥Three times	39	11.9

### 2.3 Data collection tools

In the study, data were obtained voluntarily from individuals playing tennis for recreational purposes using the face-to-face survey method. In addition to the personal information form, leisure involvement and re-participation intention scales were used as measurement tools.

**Personal information form**: The Personal Information Form includes demographic and participation-related information about the participants, such as age, gender, education level, and purpose of participation.

**Leisure involvement scale**: Leisure Involvement Scale, developed by Kyle et al. (2007) and adapted into Turkish by Gürbüz et al. (2018), was used to measure participants’ leisure involvement levels. The scale consists of 15 items across 5 sub-dimensions, with a 5-point Likert scale for responses. The Cronbach’s alpha values for the sub-dimensions range from 0.69 to 0.91 (Kyle et al., 2007). In the study by Gürbüz et al. (2018), Cronbach’s alpha values for the sub-dimensions ranged from 0.58 to 0.80.

**Re-participation intention**: This section of the tool aimed to measure participants’ re-participation intentions in recreational activities. This section used three items recommended by Zeithaml et al. (1996) and employed in Demirhan et al. (2018) study. These three items focused on participants’ intention to continue, re-participate in, and engage in different tennis activities, and were evaluated using a 7-point Likert scale. In the study by Demirhan et al. (2018), Cronbach’s alpha value for scale was 0.80.

### 2.4 Ethical approval

Before the data collection, ethical approval was obtained from the Ethics Committee of Sakarya University of Applied Sciences with the decision numbered E-26428519-050.99-149248.

### 2.5 Statistical analysis

The data were analyzed using the IBM SPSS software program. Descriptive statistics (mean, standard deviation, skewness, kurtosis) were calculated using the SPSS program. The normality of the data was checked based on the criterion that the skewness and kurtosis values fall within the ±2 range (George and Mallery, 2016). To categorize participants according to their leisure involvement levels, both Hierarchical and Non-Hierarchical Cluster Analyses were applied (Tabachnick and Fidell, 2015). In the Non-Hierarchical Cluster Analysis, K-Means Cluster analysis was used to determine the number of clusters, and in the Hierarchical Cluster Analysis, Ward’s method was employed. These methods are used when the researcher does not know the number of groups in advance, allowing the researcher to determine the groups and then analyze group membership (Çokluk et al., 2014; Nakip, 2006). To analyze the re-participation intentions of participants based on their leisure involvement levels, Univariate analysis was used.

## 3 Results

The distribution of the mean scores for the leisure involvement clusters was presented in Table 2.

**Table 2:** Final cluster centers.

Leisure involvement	Cluster 1	Cluster 2	Mean
Attractiveness	4.17	3.50	3.76
Importance	3.80	2.56	3.03
Social relationship	3.96	3.38	3.60
Identification	4.03	3.00	3.39
Self-expression	3.82	2.97	3.29
5-point Likert type			

When the results were examined, it was determined that the first cluster contained 203 participants (37.9%) and the second cluster contained 124 participants (62.1%) (Table 3). The distance between these cluster centers was found to be 2.034. Additionally, the one-way ANOVA test included in the analysis revealed that the difference between the groups was significant at the 0.01 level. As a result of the cluster analysis, participants’ leisure involvement levels were classified into two distinct groups: low and high involvement.

**Table 3:** Cluster sizes and distance between cluster centers.

Cluster	*N*	Cluster size (%)	Distances between final cluster centers	
			Cluster 1	Cluster 2
Low	203	37.9		2.034
High	124	62.1	2.034	
Total	327	100		

**Figure 1:**
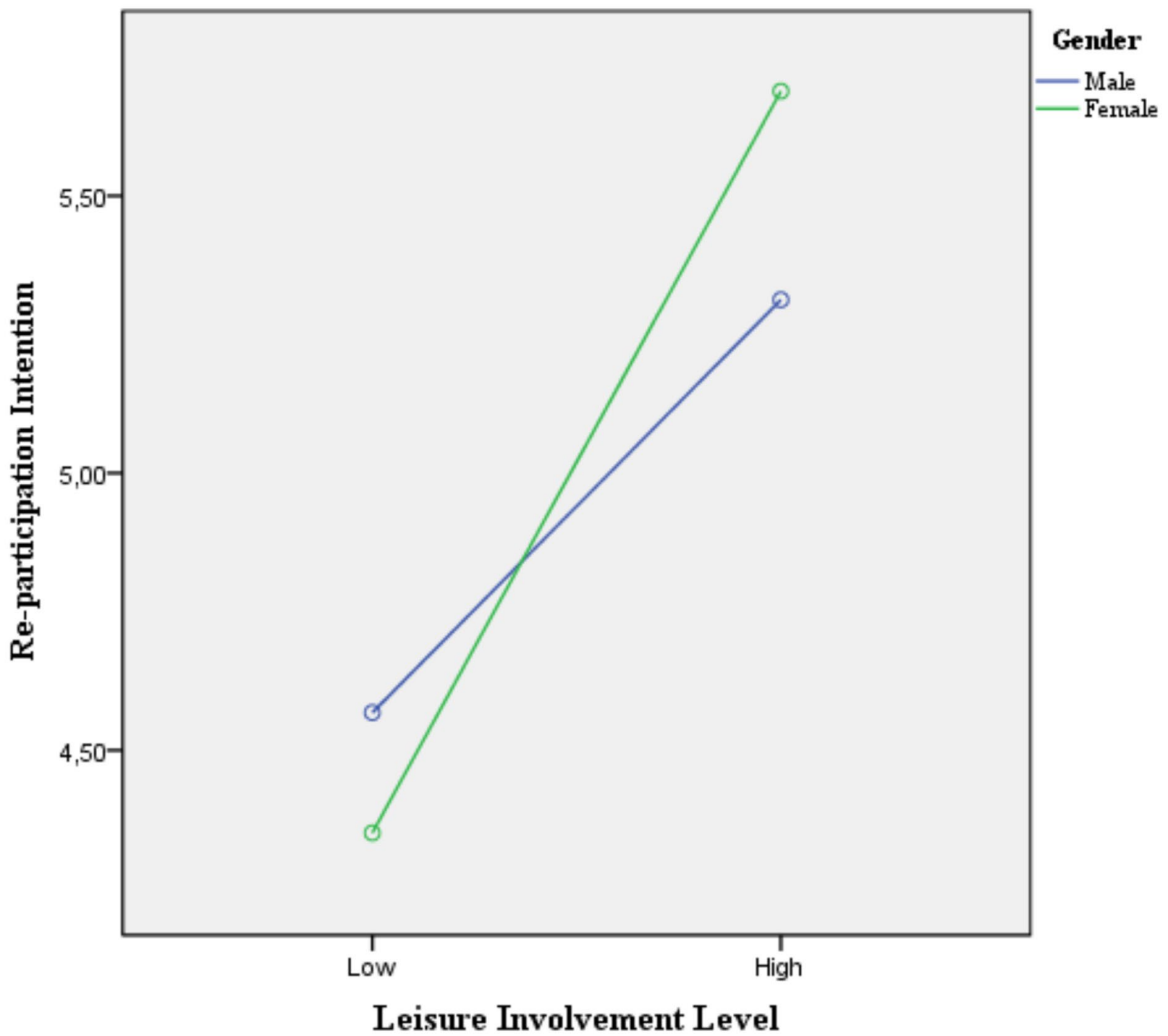
Average scores for re-participation intention of male and female with different levels of involvement.

The analysis results indicate that there was no statistically significant difference in re-participation intention based on the participants’ gender (*p* > 0.05). However, a significant difference in re-participation intention was found according to the participants’ leisure involvement levels (*p* < 0.05). Additionally, a significant interaction between leisure involvement and gender was observed in re-participation intention (*p* < 0.05; Table 4). The average scores for re-participation intention of men and women with different levels of involvement were presented in Figure 1.

**Table 4:** Results of the difference analysis on re-participation intention based on participants’ leisure involvement levels and gender.

Source	Sum of squares	Df	Mean square	*F*	*p*	*η* ^2^
Corrected model	88.163	3	29.388	23.898	0.000	0.182
Intercept	7,619.702	1	7,619.702	6,196.390	0.000	0.950
Involvement level	0.488	1	0.488	0.397	0.000	0.001
Gender	83.285	1	83.285	67.728	0.529	0.173
Involvement level*Gender	6.762	1	6.762	5.499	0.020	0.017
Error	397.193	323	1.230			
Total	8,197.111	327				
Corrected total	485.356	326				

The analysis of Figure 1 reveals that the level of involvement significantly influences re-participation intention. It was observed that the re-participation intentions of both male and female participants increased as their involvement level rose.

## 4 Discussion and conclusion

This research aimed to examine the re-participation intentions of individuals who play recreational tennis based on their low and high levels of leisure involvement. The results obtained from the analysis are discussed and interpreted in this section. According to the analysis results, a statistically significant difference was observed in leisure involvement levels based on participants’ involvement levels. This finding supports the research hypothesis. It was found that participants with a high level of involvement exhibited a higher intention to re-participate compared to those with a low level of involvement. These findings emphasize the importance of involvement levels in participation in recreational activities. Individuals with a higher level of involvement, such as in sports activities like tennis, are more likely to have stronger intentions to re-participate. This can be interpreted as an indication of these individuals’ stronger commitment to the sport and their positive perceptions of the benefits it provides.

Many studies in the relevant literature support the findings of our research. In a study by Pi et al. (2017) on hiking tourism, the effect of leisure involvement and recreational benefits on re-participation intention was investigated. The study found that both recreational benefits and leisure involvement have a positive effect on re-participation intention. Yang et al. (2019) conducted a study in the hospitality sector, examining the effect of leisure involvement and attitudes on re-participation. The research reported that leisure involvement has a positive effect on re-participation intention. Ahmad (2021) conducted a study on individuals visiting shopping malls and explored the mediating effect of quality of life on the impact of leisure involvement and place attachment on revisit intention. They found that both leisure involvement and place attachment positively influence revisit intention. Li et al. (2020) on individuals visiting the Macau Cultural Centre, the relationship between involvement, satisfaction, and behavioral intentions was examined, with a positive relationship found between involvement and behavioral intentions. Noh and Yang (2021) investigated the relationship between involvement, loyalty, and behavioral intentions in a study on individuals participating in marine recreational activities. They reported a positive relationship between involvement and behavioral intentions. These studies highlight the important role of leisure involvement in enhancing re-participation intentions across various recreational contexts, aligning with the findings of the current research.

The theory of planned behavior suggests that individuals’ intentions to re-participate in activities are strengthened when they plan the behavior in advance and possess strong motivation toward it (Burnkrant and Sawyer, 1983). The hedonic effects of recreational activities (pleasure, fun, happiness, etc.) evoke positive feelings and thoughts in individuals, which in turn triggers the desire for re-participation in these activities (Ayhan, 2023). It has been observed that individuals with high leisure involvement are satisfied with the benefits they gain from the activity and tend to continue their participation (Akçakese et al., 2024; Koo et al., 2014). Additionally, participants with high leisure involvement have been reported to experience satisfaction in physical, mental, educational, and social aspects (Bammel and Burrus-Bammel, 1996; Wankel and Berger, 1991; Chen, 2001; Bright, 2000).

The sense of satisfaction individuals gain from activities stands out as an important factor in determining their choice of leisure activities, participation decisions, and willingness to continue these activities (Park et al., 2003). Individuals with high leisure involvement experience a high level of motivation toward the activity, leading to a high-quality leisure experience. In this context, based on the research findings mentioned above, it can be stated that, similarly, the level of involvement of individuals who play tennis for recreational purposes strongly affects their re-participation intentions.

It has been determined that, for individuals who play tennis for recreational purposes, their level of involvement in tennis has a strong effect on their re-participation intention. Tennis not only provides physical benefits but also reinforces participants’ interest in the sport by offering social interaction and both physical and psychological benefits (Bum and Jeon, 2016; Spring et al., 2020; Stubbs and Werneck, 2024). In this context, a high level of involvement in tennis emerges as one of the main factors that increase individuals’ motivation to re-participate in tennis activities. The literature includes studies supporting these findings in the context of tennis players. In a study by Yang et al. (2016), which examined physical education students participating in tennis, badminton, and golf activities, it was reported that the involvement arising from high motivation had a significant impact on individuals’ re-participation intentions. In their study on adult tennis players, Casper et al. (2007) found that participation frequency, which had a significant effect on individuals’ level of involvement, was strongly related to their re-participation intentions. In a study conducted by Zeng (2020) on young tennis players, it was determined that the satisfaction of psychological needs, which had a significant impact on individuals’ level of involvement, affected the players’ motivation to participate in the activity.

### 4.1 Limitations and suggestions

While this research provides useful insights, some limitations should be considered. The findings of this study are based on individuals who play recreational tennis in Sakarya, which limits the generalizability of the study to other sports and recreational activities. To enhance the generalizability of the findings, future studies should include a more diverse population involved in various recreational sports activities. In this study, the leisure involvement variable was categorized through cluster analysis, and differences based on low and high involvement were explored. In future studies, it can be hypothesized that other unexplored variables or mechanisms may contribute to the observed relationships. Therefore, variables such as recreational benefit, recreational flow experience, recreational wellbeing, leisure satisfaction, and similar constructs could be examined, and mediation and moderation effects between these variables could be identified.

### 4.2 Recommendations for practitioners

The Ministry of Youth Sports, youth centers, and municipalities should adopt effective strategies to increase participation in recreational sports activities like recreational tennis. The Ministry of Youth Sports can promote projects that facilitate youth access to sports activities on a national level and provide the necessary funding and resources to organize sustainable events that will attract the interest of young people. The Ministry can also introduce incentive programs, such as reward systems and sports scholarships, to motivate young individuals and encourage their participation in these activities. By creating an environment that supports and encourages youth, long-term participation can be achieved.

Youth centers should maintain the engagement levels of young people by organizing regular events and competitions. For example, beginner and advanced tennis courses can provide participants with the opportunity to gain experience at different levels of sport, fostering commitment. Teamwork activities that promote socializing, friendly tournaments, and themed events can increase the enjoyment young people derive from the sport, making their participation continuous.

Municipalities should encourage participation in sports by improving the accessibility and quality of sports facilities for all members of society. Offering free or low-cost programs, particularly for children from low-income families, can support social equality. Municipalities can also raise awareness by running campaigns that highlight the physical and mental benefits of sports, motivating young people through these initiatives.

It is also important for these institutions to develop systems that track and reward participants’ achievements. Recording performance improvements and successes can increase individuals’ motivation by helping them recognize their progress and development. Overall, by fostering effective collaboration between the Ministry of Youth and Sports, youth centers, and municipalities, recreational activities can be made more attractive, and the intention of young people to re-participate can be strengthened.

## Data Availability

The raw data supporting the conclusions of this article will be made available by the authors without undue reservation.

## References

[ref1] Ahmad M. S. S. (2021). Leisure involvement and place attachment on shopping mall revisit intention: the mediating role of quality of life. Int. J. Manag. Res. Emerg. Sci. 11, 12–23. doi: 10.56536/ijmres.v11i1.124

[ref2] Ahn B. W. Kwon Y. H. (2019). The structural relationship between leisure attitude, facilitation, constraint, satisfaction, and re-participate intention among marine sports participants. J. Korea Acad. Ind. Cooper. Soc. 20, 772–779. doi: 10.5762/KAIS.2019.20.12.772

[ref3] Akçakese A. Demirel M. Yolcu A. F. Gümüş H. Ayhan C. Sarol H.. (2024). Nature relatedness, flow experience, and environmental behaviors in nature-based leisure activities. Front. Psychol. 15:1397148. doi: 10.3389/fpsyg.2024.1397148, PMID: 38903476 PMC11189019

[ref4] Ayhan C. (2023) in Tekrar katılım niyetinin yordayıcıları: Serbest zaman ilgilenimi, serbest zaman tatmini, rekreasyonel akış deneyimi ve rekreasyonel fayda. ed. Soyer F. (Ankara: Gazi Kitabevi).

[ref5] Bammel G. Burrus-Bammel L. L. (1996). Leisure and human behavior. Dubuque, IA: W. C. Brown.

[ref6] Bright A. D. (2000). The role of social marketing in leisure and recreation management. J. Leis. Res. 32, 12–17. doi: 10.1080/00222216.2000.11949878

[ref7] Bum C. H. Jeon I. K. (2016). Relationships among fun, self-esteem, and happiness of tennis players. Soc. Behav. Personal. Int. J. 44, 1619–1635. doi: 10.2224/sbp.2016.44.10.1619

[ref8] Burnkrant R. E. Sawyer A. G. (1983). “Effects of involvement and message content on information-processing intensity” in Information processing research in advertising. ed. Harris R. J. (Hillsdale, NJ: Lawrence Erlbaum Associates), 43–64.

[ref9] Casper J. M. Gray D. P. Stellino M. B. (2007). A sport commitment model perspective on adult tennis players’ participation frequency and purchase intention. Sport Manag. Rev. 10, 253–278. doi: 10.1016/S1441-3523(07)70014-1

[ref10] Chang H. H. (2017). Gender differences in leisure involvement and flow experience in professional extreme sport activities. World Leis. J. 59, 124–139. doi: 10.1080/16078055.2016.1166152

[ref11] Chang S. Gibson H. (2011). Physically active leisure and tourism connection: leisure involvement and choice of tourism activities among paddlers. Leis. Sci. 33, 162–181. doi: 10.1080/01490400.2011.550233

[ref12] Chao-Sen W. (2018). Application of TQM to road races: study on the sports involvement and willingness to re-participate. Cog. Bus. Manag. 5:1509811. doi: 10.1080/23311975.2018.1509811

[ref13] Chen Z. Y. (2001). The study of elementary teachers' leisure participation, experience in leisure benefits, and work satisfaction in Taipei County. Taipei City, Taiwan: National Taiwan Normal University.

[ref14] Chen W. H. Shao Y. L. (2006). Exploring the relationship among involvement, satisfaction, and repurchase intention of 2004 ING Taipei international marathon participants. Tpec Press 14, 146–156.

[ref15] Çokluk Ö. Şekercioğlu G. Büyüköztürk Ş. (2014). Sosyal bilimler için çok değişkenli istatistik: SPSS ve LISREL uygulamaları. 3rd Edn. Ankara: Pegem Yayıncılık.

[ref16] Demirhan O. Eskiler E. Altunışık R. (2018). Segmentation by motivational factors of fantasy football consumers and differences among segments. Phys. Cult. Sport Stud. Res. 79, 24–33. doi: 10.2478/pcssr-2018-0017, PMID: 39513035

[ref17] George D. Mallery P. (2016). IBM SPSS statistics 23 step by step: a simple guide and reference. 13th Edn. New York, NY: Routledge. ISBN.

[ref18] Gumus H. Koc M. C. Talaghir L. G. (2024). Global research trends in physical activity barriers: a study on men. Healthcare 12:2098. doi: 10.3390/healthcare12202098, PMID: 39451512 PMC11507539

[ref19] Gürbüz B. Çimen Z. Aydın İ. (2018). Serbest zaman İlgilenim Ölçeği: Türkçe formu geçerlik ve güvenilirlik çalışması. Spormetre Beden Eğitimi ve Spor Bilimleri Dergisi 16, 256–265. doi: 10.33689/spormetre.480235

[ref20] Havitz M. E. Dimanche F. (1997). Leisure involvement revisited: conceptual conundrums and measurement advances. J. Leis. Res. 29, 245–278. doi: 10.1080/00222216.1997.11949796

[ref21] Josiam B. M. Smeaton J. Clements C. J. (1999). Involvement: travel motivation and destination selection. J. Vacat. Mark. 5, 167–175. doi: 10.1177/135676679900500205

[ref22] Karasar N. (2012). Bilimsel araştırma yöntemi. Ankara: Nobel Yayınları.

[ref23] Koo S. K. S. Byon K. K. Baker T. A. I. I. I. (2014). Integrating event image, satisfaction, and behavioral intention: small-scale marathon event. Sport Mark. Q. 23, 127–137.

[ref24] Krugman H. E. (1977). Memory without recall, exposure without perception. J. Advert. Res. 17, 7–12.

[ref25] Kyle G. T. Absher J. Norman W. Hammitt W. Jodice L. (2007). Modified involvement scale. Leis. Stud. 26, 399–427. doi: 10.1080/02614360600896668, PMID: 39989647

[ref26] Kyle G. Chick G. (2002). The social nature of leisure involvement. J. Leis. Res. 34, 426–448. doi: 10.1080/00222216.2002.11949980

[ref27] Li Z. Ge Y. Su Z. Huang X. (2020). Audience leisure involvement, satisfaction, and behavior intention at the Macau science center. Electron. Libr. 38, 383–401. doi: 10.1108/EL-07-2019-0176

[ref28] Lin C. W. Feng Y. H. (2015). A study of leisure involvement, flow experience, and well-being in sport of tennis participants. J. Chiao Da Phys. Educ. 10, 1–12.

[ref29] Mitchell A. A. (1979). Involvement: a potentially important mediator of consumer behavior. Adv. Consum. Res. 6, 191–196.

[ref30] Nakip M. (2006). Pazarlama araştırmaları, teknikler ve (SPSS destekli) uygulamalar. Ankara: Seçkin Yayıncılık.

[ref31] Noh N.-J. Yang C.-H. (2021). The relationship between participation involvement, loyalty, and behavior intention of marine leisure sports participants. Ilkogretim Online 20, 1084–1092. doi: 10.17051/ilkonline.2021.03.119

[ref32] Pan S.-L. Wu H. Morrison A. Huang M.-T. Huang W.-S. (2018). The relationships among leisure involvement, organizational commitment, and well-being: viewpoints from sport fans in Asia. Sustain. For. 10:740. doi: 10.3390/su10030740

[ref33] Park B. Woo S. Ju H. (2003). Economic impact of a convention industry in Busan: the 2002 Busan international machine tool show. J. Tour. Leisure Res. 15, 31–46.

[ref34] Pi L. L. Huang C. H. Chen L. H. Kuo C. T. (2017). The impact of leisure involvement and leisure benefits on return intention of fitness walking tourism participants. J. Taiwan Sports Manag. 17, 1–22.

[ref35] Sato M. Yoshida M. Wakayoshi K. Shonk D. J. (2017). Event satisfaction, leisure involvement, and life satisfaction at a walking event: the mediating role of life domain satisfaction. Leis. Stud. 36, 605–617. doi: 10.1080/02614367.2016.1240221

[ref36] Spring K. E. Holmes M. E. Smith J. W. (2020). Long-term tennis participation and health outcomes: an investigation of “lifetime” activities. Int. J. Exerc. Sci. 13, 1251–1261. doi: 10.70252/BAHT9366, PMID: 33042385 PMC7523898

[ref37] Stone R. N. (1984). The marketing characteristics of involvement. Adv. Consum. Res. 11, 210–215.

[ref38] Stubbs B. Werneck A. (2024). The relationship between tennis participation and wellbeing: a survey of 2287 adults. Int. J. Racket Sports Sci. 6, 9–16. doi: 10.30827/ijrss

[ref39] Tabachnick B. G. Fidell L. S. (2015). Using multivariate statistics. Boston, MA: Pearson.

[ref40] Unierzyski P. (2003). Level of achievement motivation of young tennis players and their future progress. J. Sports Sci. Med. 2, 184–186, PMID: 24688282 PMC3963254

[ref41] Wankel L. M. Berger B. G. (1991). “The personal and social benefits of sport and physical activity” in Benefits of leisure. eds. Driver B. L. Brown P. J. Peterson G. L. (State College, PA: Venture Publishing).

[ref42] Yang K. Min J. H. Garza-Baker K. (2019). Post-stay email marketing implications for the hotel industry: role of email features, attitude, revisit intention, and leisure involvement level. J. Vacat. Mark. 25, 405–417. doi: 10.1177/1356766718814081

[ref43] Yang J.-Y. Uk C. H. Moon H.-W. (2016). The influence of university physical education on students’ re-participation intention in sports for all. Korean J. Growth Dev. 24, 371–376.

[ref44] Zaichkowsky J. L. (1985). Measuring the involvement construct. J. Consum. Res. 12, 341–352. doi: 10.1086/208520

[ref45] Zeithaml V. A. Berry L. L. Parasuraman A. (1996). The behavioral consequences of service quality. J. Mark. 60, 31–46. doi: 10.1177/002224299606000203

[ref46] Zeng Z. H. (2020). An exploration study of youth tennis players' participation-motivations: features and the relationships with relative elements. Sport Soc. Interdis. J. Phys. Educ. Sports 20, 1–14. doi: 10.36836/2020/1/1, PMID: 39960219 PMC12015381

